# The presence of *Candida parapsilosis* with intrathecal baclofen pump in a person with high cervical spinal cord injury; infection or colonization? A Case Report

**DOI:** 10.1038/s41394-023-00610-5

**Published:** 2023-11-30

**Authors:** John Chan, Harminder Singh, Kazuko Shem

**Affiliations:** 1 Division of Physical Medicine and Rehabilitation, Stanford University, Stanford, CA USA; 2 Division of Neurosurgery, Santa Clara Valley Medical Center, San Jose, CA USA; 3 Department of Neurosurgery, Stanford University, Stanford, CA USA; 4 Department of Physical Medicine and Rehabilitation, Santa Clara Valley Medical Center, San Jose, CA USA

**Keywords:** Fungal infection, Risk factors, Pathology

## Abstract

**Introduction:**

Intrathecal baclofen (ITB) therapy is an effective method of treating spasticity in persons with spasticity due to spinal cord injury (SCI), but complications are not rare and can include spinal fluid leaks, infection, and catheter/pump malfunction.

**Case presentation:**

This study presents information related to an adult male patient with traumatic SCI and a history of two prior ITB pump pocket infections that required removal due to pump infection. The patient then developed skin erosion over the third pump, and the fluid around the pump grew methicillin-sensitive *Staphylococcus aureus*, diphtheroids, and *Candida parapsilosis*. The patient was initially treated with antibiotics and anti-fungal medication without removal of the ITB pump. The ITB pump was eventually removed 27 months later, and the fourth pump was implanted 10 months later.

**Discussion:**

ITB pumps can be an effective treatment modality for spasticity in people with SCI; however, complications, including infection, can occur and require pump removal. This case illustrates a case of possible *Candida* colonization of the ITB pump, which was eventually removed.

## Introduction

Intrathecal baclofen (ITB) therapy is an effective method of treating spasticity in person with spinal cord injury (SCI) [1]. Though baclofen is centrally acting, it crosses the blood–brain barrier ineffectively, limiting its bioavailability when taken orally. Intrathecal baclofen allows for four times the amount of baclofen to be delivered to the spinal cord with 1% of the oral dose [2]. Thus, implanted ITB pumps allow for consistent long-term baclofen dosing and reduce the overall baclofen dose that is required to control spasticity. Although systemic side effects of baclofen are generally avoided with this approach, complications of ITB pump therapy can include pharmacological complications, spinal fluid leaks, infection, hemorrhage, catheter dislodgement/breakage, and pump problems [3, 4]. Complications of an ITB pump system have been reported to occur at a rate as high as 41% [5], and they can be categorized as either pump-related or catheter malfunction, with catheter malfunction usually occurring more frequently [4–6].

Infectious complication specifically has been reported to occur at rates ranging from less than 1% to as high as 41.7% [7, 8]. For example, Chan reported an infection rate per puncture per refill of 0.6% [9], whereas Dario et al. reported no infections in 890 refill procedures [10]. Infectious complications can also be categorized as pump pocket infection, catheter infection, and/or those causing meningitis. There are very few reports of infectious agents causing pump infections in the literature, but the most commonly reported agent is *Staphylococcus aureus* [4]. Other reported causative agents include *Pseudomonas aeruginosa, Staphylococcus epidermidis, Aspergillus terreus*, and *Enterococcus coli*, but colonization with *Candida* species has not been reported to our knowledge [8, 11–14]. The presence of gastrostomy tubes and urinary and/or fecal incontinence have been reported to be associated with a higher incidence of ITB pump infection [4, 7].

This case report describes an ITB pump in a person with SCI, which was removed presumably due to a *Candida parapsilosis* infection of the pump. This case report will discuss the assessment that was done to diagnose the ITB pump pocket infection and the management of ITB pump infection.

### Case presentation

The patient is a male in his 50s with C1–C4 tetraplegia from a motor vehicle accident in the 1980s. Similar to most persons with complete tetraplegia, he has a neurogenic bowel managed by a colostomy, a neurogenic bladder managed with a suprapubic catheter, and neuropathic pain. He also has had a chronic perineal wound for at least 10 years. His only other co-morbidity is diabetes mellitus, which was diagnosed more than 12 years ago and is controlled with metformin. His routine medications include oxybutynin, tizanidine, and senna. He developed an ITB pump infection requiring replacement in 2002 and again in 2010. He was reportedly bed-bound for about one year after each pump removal until the next pump could be implanted. His usual baclofen pump rate is 300.1 mcg/day. His spouse is his primary caregiver.

**Fig. 1:**
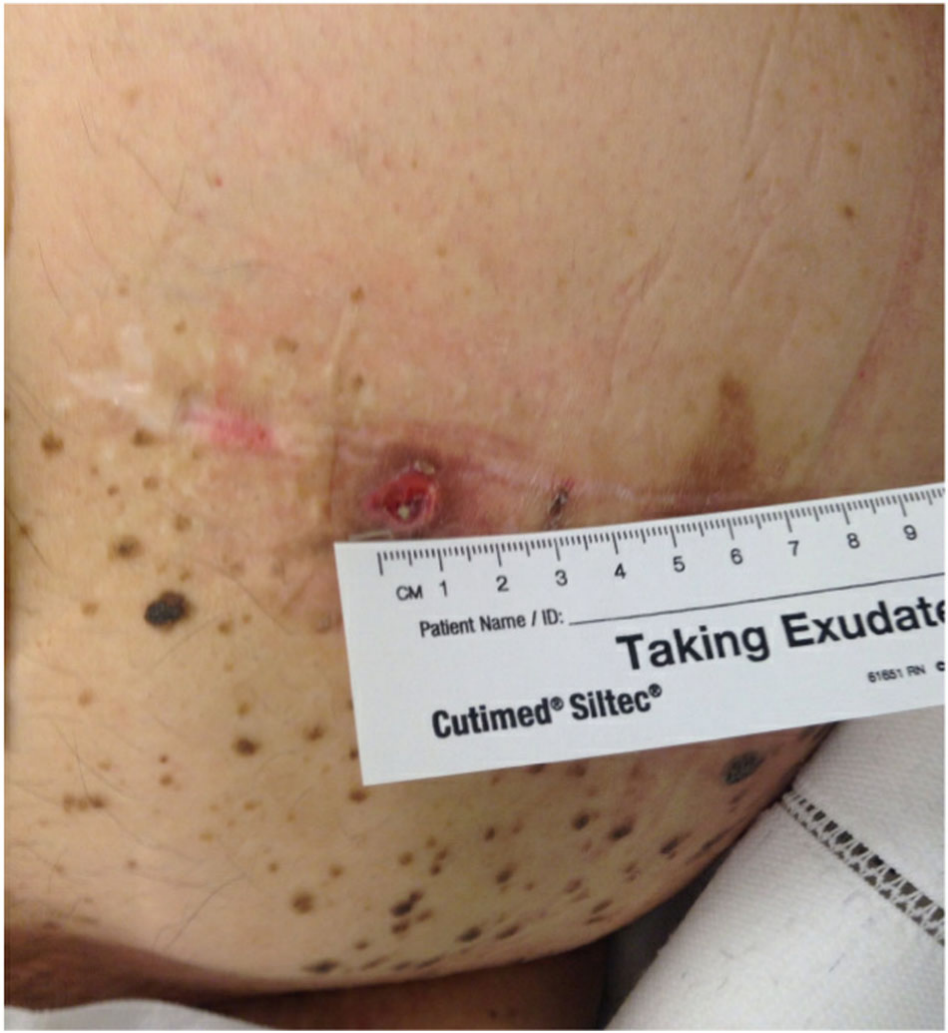
Picture of skin erosion over the intrathecal baclofen pump.

In 2018 on his third ITB pump, he was found to have skin erosion at the ITB pump site with no signs or symptoms of infection (Fig. 1). He underwent explantation and re-implantation of the pump into a deeper fat pocket in the left lower quadrant. Intraoperatively, he was found to have cloudy fluid around the pump, and cultures grew methicillin-sensitive *Staphylococcus aureus*, diphtheroids, and *C. parapsilosis*. Vancomycin powder was left behind in the pump pocket, and the patient was treated with 4 weeks of intravenous (IV) cefepime followed by ceftriaxone to cover for possible methicillin-susceptible staph aureus (MSSA) meningitis concurrently with 2 weeks of high-dose fluconazole for Candida infection and followed by trimethoprim/sulfamethoxazole for suppressive therapy.

**Fig. 2:**
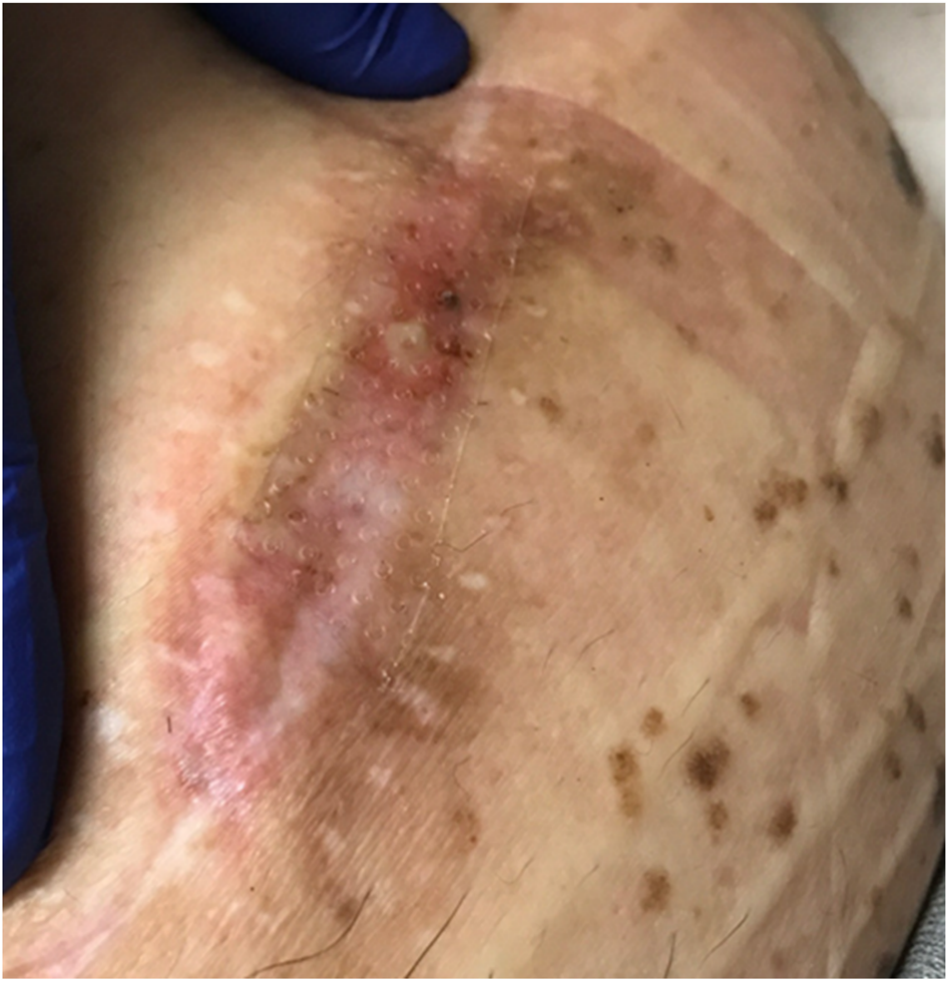
Picture of skin erosion over the intrathecal baclofen pump.

In 2019, the patient was again treated with high-dose fluconazole for suspected Candida pump infection. Neurosurgery and Infectious Disease specialists monitored him with serial C-reactive protein (CRP) measurements. CRP level initially was 3.1 mg/dl and fluctuated to as high as 6.6 mg/dl for over 26 months. Fluoro-guided lumbar puncture was attempted to obtain cerebral spinal fluid (CSF) for culture but was unsuccessful due to the patient’s diffuse idiopathic skeletal hyperostosis (DISH). In 2020, the pump was repositioned again due to concern for impending skin erosion since the pump appeared to be rising up against his skin (Fig. 2). A swab of the pump pocket at that time revealed *Candida metapsilosis*, and the patient was treated with 2 months of fluconazole.

In 2021, the patient presented with fever, hypotension, leukocytosis, and elevated CRP (24.5 mg/dl). He had no mental status changes and no meningeal signs aside from a headache, which he reported as chronic. Blood cultures were positive for Klebsiella pneumoniae. Urinalysis was concerning for possible urinary tract infection (UTI). Interventional radiology (IR) was unable to perform lumbar puncture due to complicated anatomy from DISH.

The following radiological studies were done:

- Chest X-ray was negative.
- Computer tomography (CT) of the abdomen with contrast
  1. Subtle mild heterogeneity of the kidney enhancement left greater than right, which could indicate pyelonephritis. No discrete abscess. Mild bladder wall thickening, which is stable from prior exam.
  2. Gallbladder wall thickening with mild pericholecystic inflammatory stranding without definite gallstone. This is worsened in appearance when compared with a prior study in 2019, and cholecystitis cannot be excluded.
  3. Trace bilateral pleural effusions and trace perihepatic ascites.
- Magnetic resonance image (MRI) of the brain
  1. No acute intracranial findings or abnormal enhancement.
  2. No leptomeningeal enhancement. Mild diffuse dural enhancement without dural thickening similar to prior, nonspecific, and likely within normal limits.
- Abdominal ultrasound
  1. Diffuse nonspecific gallbladder wall thickening measuring up to 7 mm. There is no additional sonographic evidence of cholecystitis. Gallbladder wall thickening may be related to hepatic adjacent disease or fluid status.
  2. Diffusely echogenic liver consistent with hepatic steatosis.

At this point, the differential diagnosis for bacteremia with Klebsiella pneumonia included:

1. Intra-abdominal source, such as *Klebsiella pneumonia*, is known to translocate from the gut, biliary tree, and genitourinary tract.
2. Cholecystitis—however, hepatobiliary iminodiacetic acid (HIDA) scan resulted in normal gallbladder imaging.
3. Pyelonephritis or complex UTI was considered given the recent treatment of UTI, but urine culture showed mixed flora.
4. Perineal wound infection—wound culture grew low levels of mixed flora.
5. Pump infection/meningitis—IR was unable to perform a fluoro-guided lumbar puncture.

As the lumbar puncture could not be completed, the decision was made to perform a side port study of the baclofen pump. Using ultrasound guidance, the pump pocket was evaluated, with no fluid collection observed. Using a 1.5-in. 25-gauge needle with sterile technique, the side port was accessed, and 20cc of CSF was withdrawn, which grew *C. parapsilosis*. The patient was started on induction therapy with both intravenous amphotericin B and oral flucytosine for six days, and the pump was removed after discussion with the patient and his neurosurgeon.

Ten months later, the patient was re-assessed for pump implantation since he continued to experience severe spasms despite being on oral baclofen, and the fourth pump was implanted without any complications. The patient remains without any symptoms or signs of pump infection since.

## Discussion

ITB therapy can be a very effective treatment option for people with SCI with severe spasticity [15]. ITB pumps are generally well tolerated by most patients, especially those who are vigilant and/or have vigilant caregivers. However, infection of the ITB pump has been known to occur in 1–42% of cases and occurs most likely around the time of implantation or replacement [4, 8]. It is especially likely in children, probably due to their more severe impairment [4]. Young children requiring intrathecal baclofen are often underweight, gastrostomy-fed, and frequently have bladder and bowel incontinence that are managed with pads: all factors contributing to higher infection risks [4, 16]. Methicillin-resistant *S. aureus* colonization and infection are also relatively common in hospitalized persons with SCI, but our patient had methicillin-sensitive *S. aureus* grow out of the fluid surrounding the ITB pump [17]. Infections may also be more likely following surgery for pump replacement rather than for new implants—a pattern that is also seen with the replacement of other implantable devices, such as pulse generator battery replacement for deep brain stimulators and cardiac pacemakers [18, 19].

Unlike pump implantation and replacement, baclofen pump refill procedures carry a very low rate of infection, with an infection rate per puncture of 0.6% [9, 10]. ITB pumps can be refilled safely, and one study reported comorbid infections such as urinary tract infections or respiratory infections do not seem to increase the risk of device contamination, but another study noted a significantly higher chance of infection in patients who have urinary and/or fecal incontinences [4, 10]. In this patient’s case, he has about one UTI per year, and he usually does not have urinary and/or fecal incontinence since he has a colostomy and a suprapubic catheter.

The most common bacteria cultured from ITB pump infections are *S. aureus* and *Staphylococcus epidermides* [13]. Because *Staphylococcus epidermitis* from skin colonization is thought to be the most common cause of foreign body infection [20], skin disinfection with an antibacterial preparation is mandatory prior to any access to the pump. Antibacterial skin preparation reduced the infection rate after pump replacement from 17.6% (3/17) to 3.3% (1/30) in one report [21].

To our knowledge, infection of the ITB pump with Candida species has not been reported. Candida infection of the central nervous system is known to occur more in neonates and those with devices like ventriculoperitoneal shunts, and the infection is usually due to *Candida albicans* [22]. Symptoms of the central nervous system due to Candida infection may present similar to acute bacterial meningitis, such as fever, headache, stiff neck, and altered mental status. In our case, the patient presented with a fever (38.8 °C) and a chronic headache but did not have altered mental status. The patient had a “stiff neck,” but he had DISH. Therefore, it was unclear to the treating team if the patient had meningitis or not.

Intrathecal baclofen pump-related infections can be serious and may cause superficial wound infection, deeper pocket infection, sepsis, or meningitis [16]. The current standard of care for deep pump infections requires pump explantation and a course of antibiotics prior to pump reimplantation [2, 16], which ultimately was done in this patient’s case. The “Turner Switch” is a newer technique of simultaneously explanting the infected pump and reimplanting a new pump on the contralateral side before initiating antibiotics. This method reduces the risk of withdrawal and length of hospitalization and eliminates the waiting period between explantation and reimplantation [23].

Infections do not always necessitate pump explantation, as some pump infections have been treated with intrareservoir instillation of antibiotics, such as vancomycin, gentamycin, and teicoplanin [12, 24]. Not all infections require intravenous antibiotics or pump explantation, with some soft tissue and even CSF infections being treated with oral antibiotics alone [14]. In our case, the patient exhibited signs of severe systemic infection, so IV antimicrobials were administered along with an explanation of the third pump.

New signs or symptoms of infection in a patient with an ITB pump necessitate a thorough evaluation of the pump. Infections in the pump pocket, reservoir, and catheter/CSF should all be considered. Initial laboratory evaluation should include white blood cell count, C-reactive protein, and sedimentation rate as markers of infection that can be subsequently used to track treatment efficacy, and the patient, in this case, report had multiple serial CRP level checks.

Lumbar puncture or side port study, as was performed in our patient, can allow for CSF analysis if meningitis is suspected. A large volume of CSF (10–20 ml) from a lumbar puncture may be required to test CSF for chronic Candida infection [22]. The pump side port, also known as the catheter access port, can be used to collect CSF for analysis if lumbar puncture is not possible, as was the case in our patient. Side port study can also be used to evaluate for catheter patency [25]. The side port is located at the “beak” of the pump and is adjacent to the connection point of the catheter to the pump. The side port does not allow access to the medication in the pump reservoir and can only be accessed with a 24- or 25-gauge needle, which is not included in the pump refill kit. This is a safety measure to prevent inadvertent administration of baclofen directly into the catheter and the patient’s intrathecal space.

In other reported cases of *C. meningitis*, the rate of positive culture is about 80% [22]. In chronic *C. meningitis*, CSF analysis can show elevated protein concentration and reduced glucose concentration; however, our patient had high glucose levels (73 mg/dl) and low protein levels (26 mg/dl). Thus, the CSF analysis was not consistent with an active Candida infection. Also, in our case, the CSF was obtained through the pump side port and not directly from the spine with a lumbar puncture. Therefore, if there was Candida colonization around the pump, it is conceivable that the CSF that was obtained was contaminated by going through the tissue around the side port.

This case is the first report of *Candida parapsilosis* that grew around the ITB pump and illustrates a challenge to diagnose pump infection vs. colonization, especially when there is a possibility of Candida infection. Because the mortality rate of chronic *C. meningitis* is high (up to 53%) [22], when Candida infection is suspected, it may still be prudent to remove the ITB pump.

### Supplementary information

CARE checklist
